# The Impact Ground Phonolite Rock’s Potassium Solubilization in Tropical Soil Depends on the Cultivated Forage Species

**DOI:** 10.3390/plants13020199

**Published:** 2024-01-11

**Authors:** Alaor Ribeiro da Rocha Neto, Renato de Mello Prado, Mara Cristina Pessôa da Cruz

**Affiliations:** Department of Soils and Fertilizers, Faculdade de Ciência Agrárias e Veterinárias, São Paulo State University (UNESP), Campus Jaboticabal, Via de Acesso Prof. Paulo Donato Castellane s/n, Jaboticabal 14884-900, Brazil; rm.prado@unesp.br (R.d.M.P.); mcp.cruz@unesp.br (M.C.P.d.C.)

**Keywords:** potassium, solubility, rock powder, cover crops, soil fertility

## Abstract

Cover crops can be used to accelerate the solubilization process of low-solubility fertilizers; thus, the aim of this study was to evaluate the potential of grasses in solubilizing potassium from phonolite rock powder. With a 2 × 5 factorial scheme, two doses of phonolite rock powder, equivalent to 0 and 8 t ha^−1^, were combined with four grass species (*Urochloa ruziziensis*, *U. decumbens*, *U. humidicola*, and *Andropogon gayanus*), besides a control treatment without any cover crop. The dry matter production of the aerial parts of the plants was evaluated at days 40 and 70 post-emergence, and then the concentration of potassium in the plants and the soil was evaluated (exchangeable, non-exchangeable, structural, and total potassium contents). In the soil, the phonolitic rock powder increased the exchangeable, non-exchangeable, structural, and total K contents, favoring the absorption of K and the production of the dry mass of the three *Urochloa*, but *U. decumbens* stood out because it promoted greater availability of K in the system compared to the cultivation of other plant species. This research proposes the inclusion of *U. decumbens* in production systems that receive phonolitic rock, constituting a sustainable strategy to improve its agronomic efficiency.

## 1. Introduction

Agricultural applications of rock powder have been studied as an alternative for potassium (K) fertilization in several countries of Africa, Europe [1], Australia, and India [2], as well as South America [3]. In addition to being a natural source of nutrients such as K, it lends itself to other purposes for the recovery of degraded areas and even as an inducer of atmospheric C sequestration processes [4].

Approximately 7–17 billion tons of rock dust and quarry by-products are generated annually as a result of mining activities globally, including in Australia, Africa, and Latin America [5], especially potassic feldspars such as orthoclase, microcline, and sanidine, and minerals from the micas group such as muscovite, vermiculite, and illite. However, their low solubility currently limits their use as potassic fertilizers [6]. It is worth noting that fine-grained phonolitic rock, in addition to feldspars, is made up of a feldspathoid (nepheline), which is more reactive in the soil, favoring, with its application, increases in the productivity of crops such as coffee [7].

A recent review of rock use in agriculture criticized issues that have constrained its use in the past and pointed out that its use promotes the production of low-cost crops, improves minimal soil fertility support, and can also contribute to several sustainable development goals [8].

One strategy that can be used to accelerate the solubilization of low-solubility minerals is the cultivation of plants with known solubilizing action by increasing the amount of K available in the soil [6,9]. Plants have the capacity to chemically change the rhizosphere both through the release of H^+^ and OH^−^ ions via the electrochemical balance and through the exudation of organic acids of low molecular weight (e.g., oxalate, citrate, and malate), which induces the dissolution of primary silicate minerals, such as potassic feldspars [2,10,11]; therefore, it is plant-dependent in the short term [10,12,13,14].

Plants can interact with microorganisms, facilitating the solubilizing action of nutrients that come from rocks [15]. Forages are associated with mycorrhiza and can be colonized by more than one species of the fungus [16], but the colonization rate for *Urochloa* is high, differing from other species such as *Megathyrsus maximus* [17]. This association increases the nutrient absorption capacity [18] despite the fact that mycorrhizal fungi cannot exude organic acids, but they can improve nutrient solubilization indirectly by stimulating other surrounding soil microorganisms through the exudation of labile C, thus increasing the local availability of nutrients in the hyphosphere and soil [19]. Therefore, K can travel across mycorrhizal hyphae membranes mediated via specific transporters [20].

Consequently, the hypothesis that low-solubility products such as rock powders can be recommended for production systems in which cover crops are used has been sustained. Generally, cover crops maintain their mineral solubilizing capacity, such as phosphorus, through the release of organic acids and enzymes because these plants have not undergone a process of genetic improvement in environments fertilized with soluble sources of nutrients [21].

In areas where cover crops are cultivated, rock powder must be applied by topdressing before sowing and then incorporated into the soil in order to exploit the solubilizing potential of plants, and the benefits for the main crop derive from the rapid liberation of K that accumulates in the dry matter (DM), which is deposited on the soil after desiccation [21], with the expectation of the release of K over the medium and long terms, thus characterizing a lasting residual effect [4,22].

Both plants and the use of rock powder are known to alter the forms and amounts of K in each soil reservoir (solution K, exchangeable K, non-exchangeable K, and structural K). There is an alternative structure to represent soil K reservoirs. The framework distinguishes between micas and feldspars as K-bearing primary minerals, based on the presence of K in interlayer positions or three-dimensional framework structures, respectively; it identifies a pool of K in neoformed secondary minerals that can include fertilizer reaction products, and it replaces the “exchangeable” K pool with a pool defined as “surface-adsorbed” K, identifying where the K is located and the mechanism through which it is held, rather than applying identification based on particular soil testing procedures [23].

Although the high growth potential of the root system of forage grasses is widely exploited to increase the sustainability of tropical agroecosystems [12], this potential has been studied little as a solubilizing agent for alternative sources of fertilizers, such as ground phonolitic rocks. Thus, it is essential to focus studies on different species of forage plants to further highlight the potential of the genetic component for the better management of phonolitic rock in pastures.

Based on the hypothesis that the use of certain plant species from the forage group with a solubilizing action can allow greater efficiency in the use of low-solubility fertilizers, this is an important finding to support a better agronomic use of this material. There may even be species with a more immediate solubilizing action when increasing the exchangeable K in the soil and others with the potential to have a greater residual effect when increasing the total K in the soil, but these hypotheses need to be proven. Hence, this research was developed with the objective of evaluating the effects of forages of the Poaceae family used as cover crops on the solubilization of phonolitic rock dust, as well as the alterations in the forms of the nutrient in the soil, on account of the plant species and product.

If this hypothesis is accepted, it will be possible for the first time to increase the agricultural efficiency of the use of ground phonolitic rock just by indicating a forage species better adapted to potassium solubilization, increasing the sustainability of agroecosystems with global implications, since it is common for soils to have low K content in different forage growing regions.

## 2. Results

**Figure 1:**
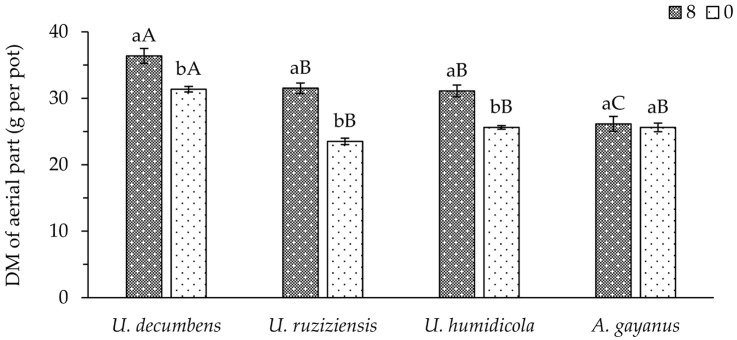
Means of dry matter (DM) produced via four cover crop species of the Poaceae family using doses (0 and 8 t ha^−1^) of phonolite rock powder. Lowercase and uppercase letters compare the mean values of doses and plant species, respectively, according to the Student–Newman–Keuls test at a 5% probability level.

The production of DM significantly differed according to the use of phonolite rock powder and the species of cover crop (Figure 1). The highest production was observed for *U. decumbens* when 0 or 8 t ha^−1^ of the fertilizer was used in comparison to other species. However, the application of 8 t ha^−1^ achieved increased DM production for all species except *A. gayanus*.

**Figure 2:**
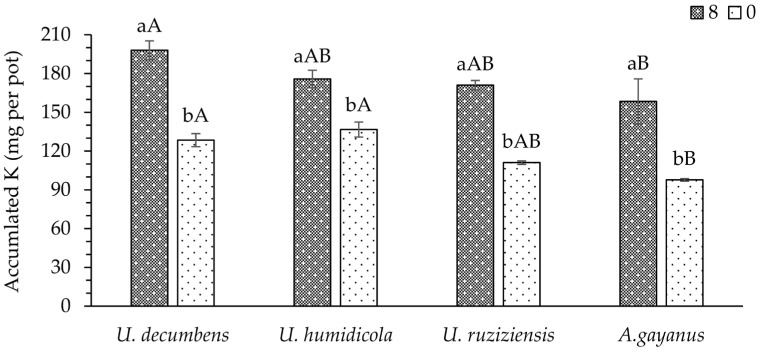
Mean values of accumulated K in the aerial parts of the species used as cover crops. Lowercase and uppercase letters compare the mean values of doses and plant species, respectively, according to the Student–Newman–Keuls test at a 5% probability level.

Regarding the accumulated K in the aerial parts of the plants, no interaction was observed between doses of phonolite rock powder and cover crop species (Table A1). Nevertheless, the plants accumulated higher amounts of K in the aerial parts with an application of 8 t ha^−1^ of the fertilizer (Figure 2). Among the plant species, *U. decumbens* was verified to have accumulated more K in its tissues in comparison to *U. ruziziensis* and *A. gayanus* while being statistically similar to *U. humidicola*. The smallest amounts of K accumulated in the DM of the plants’ aerial parts were found in *U. ruziziensis* and *A. gayanus*.

**Figure 3:**
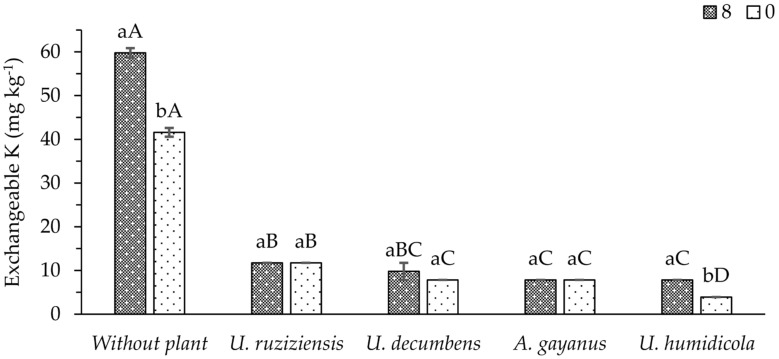
Mean values of the exchangeable K remaining in the soil via cropped species and doses (0 and 8 t ha^−1^) of phonolite rock powder. Lowercase and uppercase letters compare the mean values of doses and plant species, respectively, according to the Student–Newman–Keuls test at a 5% probability level.

An interaction between phonolite doses and cover crop species was observed for the exchangeable K remaining in the soil after cutting the plants. In the treatment without cover crops, the application of phonolite rock powder increased the pre-existing content of exchangeable K in the soil (Figure 3). However, in the presence of cover crops, very low levels of this element remained in the soil, regardless of the application of the fertilizer, for most species evaluated. Only for *U. humidicola* was higher exchangeable K content maintained in the soil when 8 t ha^−1^ of the fertilizer was applied.

The forms of non-exchangeable, structural, and total K in the soil increased with the application of phonolite rock powder, but only the total K content was altered via the cover crops. As shown in Table A1, the total K contents in the soil were reduced in the presence of plants in the system, but no effects were observed regarding the species.

**Figure 4:**
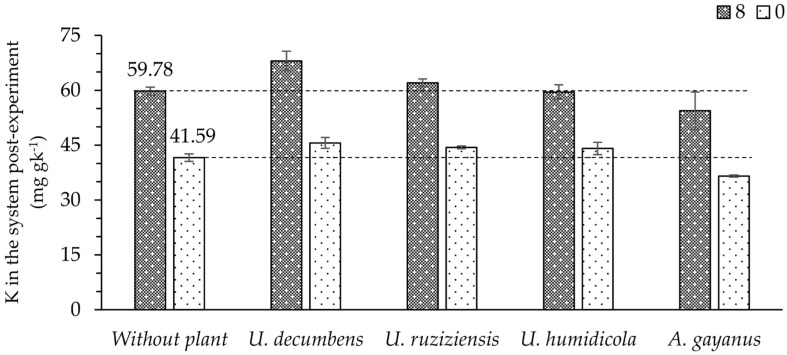
Total available K in the system at the end of the experiment according to the cropped species and doses (0 and 8 t ha^−1^) of phonolite rock powder application.

The potential of the plants to solubilize K derived from the phonolite rock powder throughout the experimental period is presented in Figure 4. When 8 t ha^−1^ of the fertilizer was applied, a higher amount of K in the system in which *U. decumbens* was cultivated was observed in comparison to the other species at the end of the experiment, while at the dose 0 t ha^−1^, no significant difference was observed for any of the cropped species.

Considering the total available K in the system after cultivating *U. decumbens* with 8 t ha^−1^ of phonolite rock powder, 68.02 mg kg^−1^ of this nutrient was observed, while in the soil without any plant used as a cover crop, a total of 59.78 mg kg^−1^ was registered. The difference is equivalent to 25 kg ha^−1^ of additional K_2_O made available in the system due to the presence of this species throughout the 70 days of the experiment.

## 3. Discussion

Considering the short-term cultivation conditions and low K availability in the soil used in this study, according to the criteria described in [24], the increased DM production of most of the evaluated species could be related to the supply of K via fertilization with phonolite rock powder even though this is considered a low-solubility source. Forage plants display mild responses to potassic fertilization; however, according to [25], such a low response occurs when medium to high K content exists in the soil. Otherwise, this fertilization results in higher DM production, as was demonstrated (Figure 1).

**Figure A1:**
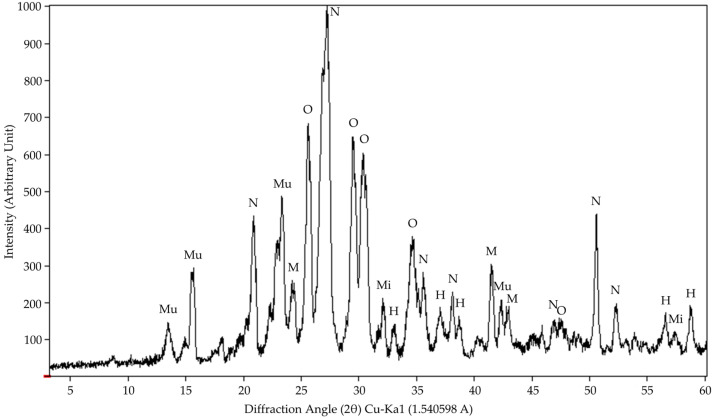
Diffractography of the mineralogical phases present in the composition of the phonolite rock powder used in the experiment. O—orthoclase; N—nepheline; Mu—muscovite; H—hyalophane; M—microcline; Mi—microsommite.

Furthermore, another factor that could benefit the increase in plants’ DM production would be the “corrective” effect of ground rock in increasing the pH value of the soil, but this did not occur in the experiment since there was no significant difference for this variable (F test, *p* ≥ 0.05). This was due to the fact that the material used did not contain a strong base of sufficient quantity to neutralize the soil acidity (Figure A1). Added to this is the fact that the soil was administered a limestone application, and the pH value of the soil was not a limiting factor for the plant growth.

The K accumulated in the aerial parts of the plants followed the same pattern of the soil availability for this nutrient. This capacity of forages to absorb K is little understood with the use of sources coming from rock, but this pattern was similar to that reported with the use of soluble sources of the nutrient seen in these forages, such as *U. decumbens* [26] and of *U. ruziziensis* [27].

Potassium ions are available to plants from the soil; therefore, their increased concentration (Figure 3) due to phonolite fertilization shows that the content of soluble K in the citric acid (2%) of the product is capable of increasing the soil’s concentration of exchangeable K. In soils in which the load is predominantly associated with organic matter or kaolinite, as was the case for the soil used in the present work, according to [23], exchangeable K determinations reliably quantify surface-adsorbed K that is bioavailable to plants. The release of K from the phonolitic rock into the soil probably occurs due to the fact that part of the composition of this rock is made up of nepheline (Figure A1), which is more reactive in the soil [7].

There have been reports that the use of phonolitic rocks (8% K_2_O) also increased the concentration of exchangeable K in the soil in forage plants grown in the field [28]. However, in both studies, this increase in exchangeable K was not enough to reach the critical level of the element in the soil for forage, as indicated by [29] (51–80 mg dm^−3^ K). The study by [28] analyzed a phonolitic rock dose (5 t ha^−1^) lower than the one used in the current work (8 t ha^−1^), and it was not enough to increase the forage dry mass production, possibly due to the lower dose used. The performance of *U. decumbens* stood out, as it was one of the forages that absorbed the most K, and at the same time, its cultivation provided the highest exchangeable K content in the soil. This may indicate that this forage has an important immediate effect on the K solubilization of the ground rock studied.

Plants also act by altering the available forms of K in the soil due to interaction with rhizospheric soil [30]. Based on the results observed in this study, in which the presence of plants systematically reduced the exchangeable K in the soil, even with the application of 8 t ha^−1^ of phonolitic rock powder, this effect may be related to the mobility of this element in the soil, which favors ion–root contact, as well as the high demand of the species and, according to [27], the very low levels of K reserves in the soil used in this experiment.

The increased concentrations of non-exchangeable and structural K in the soil due to phonolite fertilization is related to the presence of minerals containing these forms of K in the rock studied (Figure A1). Both forms of K are contained between layers and within crystalline structures of minerals, especially in the groups of micas and feldspars [31], such as muscovite, orthoclase, microcline, and nepheline. Thus, the total K that exists in the soil, which comprises the sum of its exchangeable, non-exchangeable, and structural forms, must also increase in concentration when rock powder is applied.

Since no alteration was found regarding the non-exchangeable and structural forms of K in the soil in the presence of the different cover crop species used in this study (Table A1), the reduction in the total K of the soil in the presence of the plants could have been strictly connected with the reduced contents of exchangeable forms of the nutrient due to the plants’ absorption without any differences being observed among the species.

The solubilizing effect that plants can exert in the rhizosphere region is attributed to the liberation of H^+^ ions and organic acids, which increase the environment’s acidity and induce the dissolution of existing minerals [10,11,12,13,14,15,16,17,18,19,20,21,22]. In addition, *Urochloa* is highly associated with mycorrhiza [17] since it stimulates solubilizing microorganisms in the rhizosphere [19], which favors the release of K that is transported inside the cells’ hyphae via specific transporters [20].

The effect observed for *U. decumbens* (Figure 4) may be related to the strategic characteristic of this species in adapting to different levels of soil fertility, changing the conditions of the rhizosphere [32]. Therefore, non-exchangeable forms of K that exist in phonolitic rock powder may be unstable in the plant rhizosphere due to acidification, as previously reported [32]. The correlation found in this study between the accumulations of K via *U. decumbens* and both structural and non-exchangeable forms of K in the soil, respectively represented by the R-values of 0.85 and 0.68 (test F *p* < 0.01), revealed this species’ ability to absorb these forms of K in the soil when phonolite powder is applied as a fertilizer. The strong ability of *Urochloa* to utilize non-exchangeable K from the soil has been reported by [27,33].

Non-exchangeable K in the soil is unstable, and it can maintain equilibrium with the K found in a soil solution through increased acidity. This is because potassium feldspars can be dissolved through an already-known plant-induced reaction via acidification in the rhizospheric region, and species with solubilizing actions can, in turn, absorb the released K that accumulates it in their tissues [10,22,31]. In addition, the solubilizing effect of potassic feldspar minerals via *Allium porrum* cultivated in washed sand has been reported after the application of syenite and phlogopite (potassic rocks), which evidences that, even when growing in an alternative substrate other than soil, plants release organic acids and solubilize K derived from sources of low solubility in environments with a minor availability of this nutrient [22]. Thus, by applying sources with these forms of K in the presence of plants such as *U. decumbens*, an increased concentration of K might be observed in the system due to the solubilizing effect. The result observed only in treatments containing phonolite powder indicates that increased concentrations of low-solubility forms of K in the soil enlarge the contact of the plant’s rhizosphere and that higher amounts of K are solubilized and absorbed.

Considering that oxisols are commonly poor in minerals containing K, in minor concentrations of these compounds in the coarsest fractions of the soil, such as sand [22], the contact of these particles with soil acids and plant roots is low, and no effect is observed for the minerals in the soil without an input of fertilizers, as was observed in this study.

From the results, differences in the performance of forages, with some practical implications for better efficiency in the use of the area that received the phonolitic rock, were clear. If, after forage cultivation, other species with shorter growth cycles or more demand for K are to be included, previous cultivation of *U. decumbens* may be indicated right after the application of ground rock since it performs a slightly more immediate action to increase the available K content in the soil. However, in the future, if the fertilized area is intended for use with other species, prior cultivation with *U. decumbens* may be indicated soon after the application of ground rock since it stands out in providing higher (total) K content in the soil and could possibly have a higher residual effect on the production system, justifying the use of long-cycle crops after forage cultivation.

Our discovery indicates the great potential of *U. decumbens* forage for K solubilization in rock since it can achieve the release of the element in soil equivalent to 25 kg ha^−1^ of K_2_O during a cultivation period of 70 days (or 130 kg ha^−1^ of K_2_O per year), which constitutes an agronomically reasonable release. This indicates that the importance of these milled silicate rocks for agriculture is underestimated, as was already warned in a recent review by [8], but with the advancement of research, this condition should be revised. It is important to carry out further studies so that other benefits of this product can be evaluated, such as the release of Si, because reports have indicated that not only is it a beneficial element but it can also favor the growth of forages [34,35], as well as its ability to sequester atmospheric CO_2_ [4].

## 4. Material and Methods

### 4.1. Experiment Location

This study’s experiment was conducted in a greenhouse of the soil and fertilizer department of the Faculty of Agrarian and Veterinarian Sciences at Unesp, Jaboticabal campus, located in the State of São Paulo, Brazil, between September 2019 and February 2020.

### 4.2. Treatments and Experimental Design

With a completely randomized factorial 2 × 5 scheme, two doses of phonolite rock powder (equivalent to 0 and 8 t ha^−1^) were combined with four grass species of the Poaceae family (*Urochloa ruziziensis*, *U. decumbens*, *U. humidicola*, and *Andropogon gayanus*), in addition to a control treatment without any cover crop. The treatments were assembled with four replicates each.

### 4.3. Soil and Phonolitic Rock Data

Samples of a clayey oxisol were collected from the 0–20 cm layer in an area previously cultivated with *Pinus* spp. for use in this experiment. The soil used was air-dried, sieved (6 mm mesh), rigorously homogenized, and sampled for chemical characterization according to the methods described in [36]. The obtained results were a pH (CaCl_2_) of 3.8, organic matter at 34 g dm^−3^, P-resin at 12 mg dm^−3^, K at 1.4 mmol_c_ dm^−3^, Ca at 6 mmol_c_ dm^−3^, Mg at 2 mmol_c_ dm^−3^, H + Al at 85 mmol_c_ dm^−3^, Al at 12 mmol_c_ dm^−3^, SB (Ca + Mg + K) at 9.4 mmol_c_ dm^−3^, CEC [SB + (H + Al)] at 94.4 mmol_c_ dm^−3^, and a V% [(SB × 100)/CEC] of 10.

The product used as a source of K in this experiment was phonolite rock powder extracted from the highlands of Poços de Caldas, Brazil, from igneous rocks of an alkaline affiliation with a mineralogical composition of 70–75% feldspars and of 20–25% feldspathoids (nepheline). The product was analyzed, 15.9% of the total K_2_O was obtained by means of complete perchloric–hydrofluoric digestion [37] and approximately 0.1% of soluble K_2_O in citric acid 2% at a ratio of 1:100, according to the official method for fertilizer analysis described in [38]. In addition to potassium, the phonolitic rock contained 25% Si, 9.1% Al, 3.1% Na, 1.0% Ca, 0.15% Mg, 0.02% P, 0.15% Mn, 0.002% Zn, and 0.04% P. The granulometry of the product indicated that 80% of the material passed through a sieve with a mesh size of 0.074 mm.

A complementary analysis was carried out using a 5 g sample of phonolitic rock powder (a 0.074 mm sieve) that was subjected to the X-ray diffraction (XRD) technique to identify the mineralogical phases present in the material (Figure A1). The equipment used was a *RIGAKU* Miniflex II System X-ray Diffractometer (Tokyo, Japan) operating with CuKα radiation (0.1540562 nm) at 30 kV and 15 mA and an angular range from 3 to 60 °2θ at a step of 0.02 °2θ s^−1^, and the diffraction patterns were interpreted using Match Software version 3.15 with the COD platform as a database [39].

### 4.4. Experiment Development

The water retention capacity of the soil was determined using metal cylinders that were filled with dry soil and placed in a recipient with water until the soil became saturated [40]. After the gravitational water was drained and the dry soil’s mass at field capacity was identified, the total retention was determined so that, during the experiment, a 50% water retention level could be adopted for plant irrigation. Bulk density (d = 1.26 g cm^−3^) was assessed using the mean value obtained by weighing three soil samples (1 dm^3^) in a measuring cylinder. Masses equivalent to 2.7 dm^3^ of soil that was used in each experimental unit were weighed and transferred to plastic bags to be further mixed with acidity improvers and the phonolite rock powder.

Based on the chemical characterization of the soil, quantities of 2.99 g of CaCO_3_ p.a. per pot and 1.45 g of 4MgCO_3_.Mg(OH)_2_.5H_2_O p.a. were weighed in order to increase the degree of base saturation to 40%, maintaining a Ca:Mg relation of 2:1. Then, 10.8 g of phonolite rock powder (equivalent to 8 t ha^−1^) was also added to each pot. These products were initially mixed into 80 cm^3^ of soil in their respective treatments and carefully merged in order to guarantee higher homogenization of the final blend. Subsequently, the blend was distributed in pots with a total capacity of 3.4 L. The bottom of these experimental units was covered with a plastic screen in an attempt to avoid losing soil. The dimensions of the pots were 18, 13, and 18 cm, respectively, in superior diameter, lower diameter, and height.

At the time of the installation of the experiment, from the total volume of water calculated to reach 50% of the water retention capacity, 100 mL was subtracted and then further added with the nutrient solution used in plant fertilization. From the remaining volume, two parts were applied in the place below the experimental unit in order to moisten the soil via capillarity, thus avoiding excessive compaction, while the remaining part of the water was added to the soil surface. Then, 100 mL of a nutrient solution was applied; it contained 100 mg dm^−3^ of N, 80 mg dm^−3^ of P, 20 mg dm^−3^ of S, 1.5 mg dm^−3^ of Zn, 1.0 mg dm^−3^ of Cu, 0.5 mg dm^−3^ of B, and 0.05 mg dm^−3^ of Mo. The sources used for this nutrient solution were (NH_4_)_2_SO_4_, NH_4_H_2_PO_4_, ZnSO_4_.7H_2_O, CuSO_4_.5H_2_O, H_3_BO_3_, and (NH_4_)_6_Mo_7_O_24_.4H_2_O. After the solution was applied, the soil was incubated for seven days; the pots were covered with a paper sheet, and the water was added when the evaporation exceeded 50 mL.

After incubation, seeds were sown at a depth of 5 mm, and the pots remained covered until the emergence of seedlings. Thinning was performed between days 6 and 8 post-emergence, and four plants were kept in each pot. Throughout the growing period, two cover fertilizations were applied at days 30 and 60 post-emergence, using 100 mL of a solution containing 100 mg dm^−3^ of N-urea. Water repositioning followed the same protocol adopted during incubation.

### 4.5. Experiment Evaluations

Two cuts were made in the aerial parts of the plants at days 40 and 70 post-emergence, with the first cut performed at a 10 cm height in relation to the base and the second cut made at the soil surface. The samples were washed in tap water, then a biodegradable, neutral, phosphate-free detergent solution (1 mL L^−1^ of water), and then deionized water. Subsequently, the samples were dried in an oven (65–70 °C) until achieving a constant weight so that DM production could be measured. The dried samples were milled and incinerated to determine the K in the tissue according to the methodology of [41].

After the final cutting of the plants, the soil was removed from the experimental units and sieved (a 4 and 2 mm mesh) for root separation, rigorously homogenized, and sampled to determine the exchangeable, non-exchangeable, and total K using the extractors NH_4_Cl 1 mol L^−1^ [36], heated HNO_3_ 1 mol L^−1^, and perchloric–hydrofluoric digestion [37], respectively. The non-exchangeable K was considered the content obtained with nitric acid subtracted from the content obtained with ammonium chloride (exchangeable), and the structural K was considered the total K content of the soil after both the exchangeable and non-exchangeable contents were subtracted, as suggested by [37].

The solubilization of K via the action of the plants was defined based on calculations that took into account the quantity of available total K in the system at the end of the experiment, represented by the remaining exchangeable K in the soil, in addition to the K accumulated via the plants.

### 4.6. Statistical Analysis

The obtained data were subjected to a variance analysis using the F test, and in cases with significant effects of the isolated factors or their interaction, means were compared using the Student–Newman–Keuls test at a 5% probability level. In addition, a correlation analysis was performed using the accumulated K, non-exchangeable K, and structural K in order to verify the capacity of the plants to absorb the nutrient in low-soluble forms from the soil.

## 5. Conclusions

In the soil, phonolitic rock powder increased the exchangeable, non-exchangeable, structural, and total K contents, favoring the absorption of K and the production of dry mass of the three *Urochloa*, but *U. decumbens* stood out because it promoted a greater availability of K in the system compared to the cultivation of the other plant species.

Based on this research, the inclusion of *U. decumbens* in monoculture or even intercropped production systems in areas treated with phonolitic rock is proposed. This approach constitutes a sustainable strategy to improve the agronomic efficiency of phonolitic rock.

## Data Availability

The data that support the findings of this study are available on request from the corresponding author, Alaor R. R. Neto.

## Appendix A

**Table A1:** Mean values of dry matter (DM) of aerial parts of plants and accumulated K in both plants and soil in relation to doses of phonolite rock powder and cover crop species.

Phonolite Doses	Plant		Soil			
	DM Aerial Part	K Accumulated	K Exchangeable	K Non-Exchangeable	K Structural	K Total
(t ha^−1^)	(g per pot)	(mg per pot)	(mg kg^−1^)			
0	26.52	118.47 b	14.57	28.89 b	1053.55 b	1097 b
8	31.29	175.81 a	19.39	38.22 a	1184.45 a	1242 a
Species						
Without plant	-	-	50.68	31.48	1218	1300 a
*U. decumbens*	33.88	163.25	8.80	34.70	1124	1167 b
*U. humidicola*	28.35	156.21	5.87	34.64	1082	1123 b
*U. ruziziensis*	27.51	141.05	11.73	32.27	1076	1120 b
*A. gayanus*	25.88	128.05	7.82	34.68	1095	1138 b
Doses	75.61 **	109.97 **	96.40 **	41.03 **	14.51 **	17.42 **
Species	40.09 **	8.29 **	1197.45 **	0.92 ns	2.31 ns	3.76 *
Doses × Sp.	8.02 **	1.39 ns	48.78 **	2.08 ns	0.07 ns	0.07 ns
CV (%)	5.4	10.5	9.1	13.7	9.7	9.4

Means followed by the same lowercase letter in columns are statistically similar according to the Student–Newman–Keuls test at a 5% probability level. F test: (ns) not significant; * significant at 5%; ** significant at 1%. CV: coefficient of variation.
